# Steady-state 4D flow using double gating: a healthy volunteer study

**DOI:** 10.1186/1532-429X-17-S1-P410

**Published:** 2015-02-03

**Authors:** Stanislas Rapacchi, Yutaka Natsuaki, Paul J Finn, Gerhard Laub, Daniel B Ennis, Peng Hu

**Affiliations:** 1 Radiology, UCLA, Los Angeles, CA, USA; 2 Siemens, Los Angeles, CA, USA

## Background

4D Phase-Contrast MRI ("4D Flow") has become a choice modality for assessment of flow patterns in complex vasculatures, such as congenital heart diseases. For thoracic imaging, the technique relies on ECG-triggering and an imaging navigator to compensate for breathing motion [Markl-JMRI2007]. The acquisition of the navigator requires switching off the 4D flow acquisition, thus breaking the steady-state and missing a phase in the cardiac cycle. We propose to use double gating from ECG and a pressure-driven belly-belt to monitor breathing motion while maintaining continuous steady-state.

## Methods

We compared in 8 healthy volunteers on a 1.5T MRI: the double-gated 4D Flow (BEL4D) with the navigator gating 4D Flow (NAV4D) and a free-breathing double-gated high temporal resolution 2D PC-MRI (BEL2D). 4D Flow was oriented in sagittal view with parameters: VENC=200cm/s, TE/TR=3.1/5.2ms, 2x2x2.2mm^3^, 67ms res., FOV: 320x200x97mm^3^, 20% slice oversampling, GRAPPA 2 (ext. ACS), duration of scan: 8-18min. 2D PC-MRI was acquired with only through-plane encoding, with identical parameters but for: TE/TR=2.7/4.4ms, 2x2x5mm^3^, 27ms res., duration of scan <1min. Navigator window was set to ±5mm with drift correction and belt threshold was set at 40% of maximum amplitude. The net flow and the peak flow were measured in the aortic root (AO), the right and left pulmonary arteries (RPA & LPA) and the main pulmonary artery (MPA). A repeated measurement ANOVA with Bonferroni correction was performed for statistical analysis.

## Results

**Figure 1:**
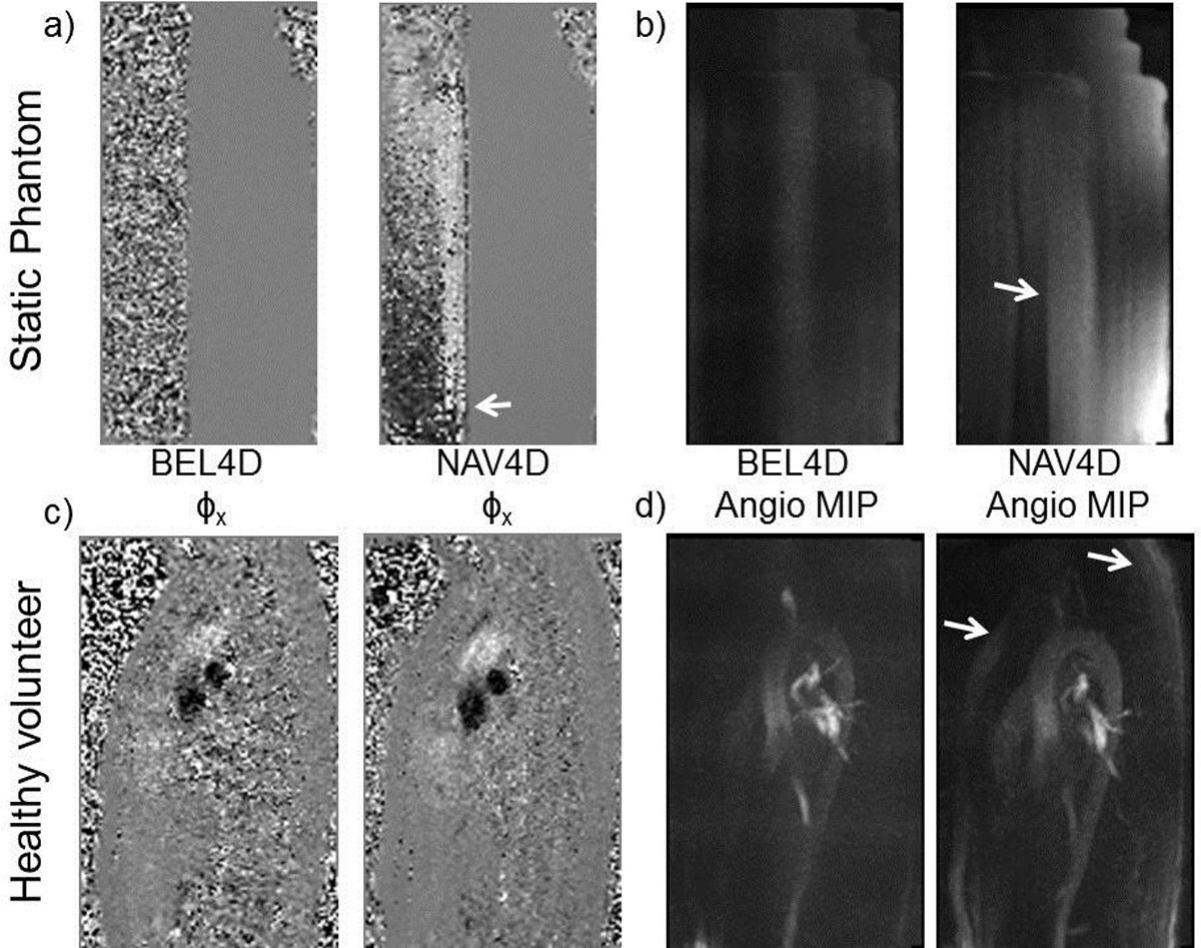
**Navigated 4D Flow (NAV4D) exhibits phase artifacts (arrows) observed on phase images (left) as well as complex difference images (right).** These artifacts are reduced with steady-state double-gating (BEL4D). Image pairs share the same window level.

Figure 1 shows typical phase artifacts, particularly observed during the first phases acquired after the navigator. The artifacts are easy to observe on the complex difference image typically employed for angiography. Double gating allows the reduction of these artifacts at the cost of lower SNR due to lower average signal. Using double-gating, it was possible to acquire systematically an extra cardiac phase.

Quantitatively for both net and peak flow and for all vessels measured, there was no statistical differences (P>0.5) between the 3 techniques. The net flow mean difference between BEL4D, NAV4D and BEL2D was under 15mL/s in larger vessels (AO&MPA) and under 17ml/s in smaller vessels (RPA&LPA). Larger mean differences were observed when comparing the MPA to the sum of RPA and LPA, up to 23 mL/s for BEL2D.

## Conclusions

Using the double-gated 4D Flow technique, we found equivalent flow quantification as with the navigator, but with reduced phase artifacts and a complete set of cardiac phases for flow assessment. Double-gated 4D Flow is an appealing technique, reproducible and reliable at studied spatial resolution. Application in a patient population is commended, with the benefit of the extra cardiac phase that can prove critical in the diagnosis of abnormal flow patterns.

## Funding

N/A.

**Table 1:** Average net flow measured by the 3 techniques in 4 main vessels.

Net Flow (mL/s)	AO	MPA	RPA	LPA	MPA-(RPA+LPA)
BEL 4D	101 ± 10	100 ± 10	38 ± 4	48 ± 9	-10 ± 9

NAV 4D	110 ± 18	92 ± 13	55 ± 13	46 ± 8	14 ± 7

BEL 2D	97 ± 11	86 ± 11	42 ± 5	37 ± 9	7 ± 5

AO: aorta; MPA: main pulmonary artery; RPA: right pulmonary artery; LPA: left pulmonary artery.

